# Improved prevention and treatment strategies for differentiation syndrome contribute to reducing early mortality in patients with acute promyelocytic leukemia

**DOI:** 10.1038/s41408-024-01074-y

**Published:** 2024-07-15

**Authors:** Qian Wu, Xiaofei Yang, Jingren Zhang, Mengxing Xue, Xueqing Dou, Zheng Ge, Yifei Chen, Weiying Gu, Weimin Dong, Hongying Cao, Naike Jiang, Xuemei Sun, Zefa Liu, Jinning Shi, Hui Chen, Cixian Zhang, Fengling Min, Hongli Sun, Xiaoli Qian, Hongjian Yuan, Yuan Feng, De-Pei Wu, Suning Chen

**Affiliations:** 1 National Clinical Research Center for Hematologic Diseases, Jiangsu Institute of Hematology, the First Affiliated Hospital of Soochow University, Suzhou, PR China; 2 Institute of Blood and Marrow Transplantation, Collaborative Innovation Center of Hematology, Soochow University, Suzhou, China; 3 Zhongda Hospital Southeast University, Nanjing, PR China; 4 Jiangdu People’s Hospital of Yangzhou, Yangzhou, PR China; 5 The First People’s Hospital of Changzhou, Changzhou, PR China; 6 Changzhou No.2 People’s Hospital, Changzhou, PR China; 7 Jiangsu Province Hospital of Chinese Medicine, Nanjing, PR China; 8 Xinghua City People’s Hospital, Taizhou, PR China; 9 The Affiliated Jiangning Hospital of Nanjing Medical University, Nanjing, PR China; 10 Yancheng NO.1 People’s Hospital, Yancheng, PR China; 11 Xuzhou Central Hospital, Xuzhou, PR China; 12 Affiliated Hospital of Yangzhou University, Yangzhou, PR China; 13 Wuxi People’s Hospital, Wuxi, PR China; 14 Taizhou Second People’s Hospital, Taizhou, PR China; 15 Zhenjiang First People’s Hospital, Zhenjiang, PR China

**Keywords:** Acute myeloid leukaemia, Immunological disorders

To the Editor,

With the introduction of differentiating agents, such as all-trans retinoic acid (ATRA) and arsenic trioxide (ATO), the outcomes of patients with acute promyelocytic leukemia (APL) have significantly improved [1, 2]. However, there are still many early deaths, especially among those at high risk [3, 4]. In our retrospective study of 570 patients with APL, early mortality was 7.54% [5]. We observed unexpected and preventable early deaths, mainly caused by steroid-unresponsive differentiation syndrome (DS) and lethal hemorrhage.

Prophylaxis against DS with steroids is recommended for patients with elevated white blood cell (WBC) counts [6, 7]. However, the optimal scheme remains controversial. Additionally, patients with DS insensitive to steroids typically have a poor prognosis, highlighting the necessity for improved management strategies in this area. In this study, patients received varying doses of dexamethasone for DS prophylaxis and the efficacy and safety of ruxolitinib were explored as a second-line treatment for DS, with the goal of reducing early mortality in APL patients.

This Phase 3 multicenter single-arm APL-01 trial (NCT04446806) enrolled previously untreated patients with APL. The present analysis was conducted in September 2023, with a median follow-up of 34 months. All patients received induction treatment with ATRA (25 mg/m^2^) along with either intravenous ATO (0.16 mg/kg capped at 10 mg) or oral tetra-arsenic tetra-sulfide (As4S4) formula named the Realgar-Indigo naturalis formula (RIF) (60 mg/kg) [8]. Bone marrow aspirate was evaluated after recovery of blood cell counts. The prevention regimen for DS was based on the WBC count at presentation and after the initiation of ATRA, which included cytoreductive agents and dexamethasone (Fig. 1A**)**. The diagnosis and grading criteria for DS were consistent with those of the PETHEMA group [9], detailed descriptions are available in the Supplementary Appendix.

**Fig. 1:**
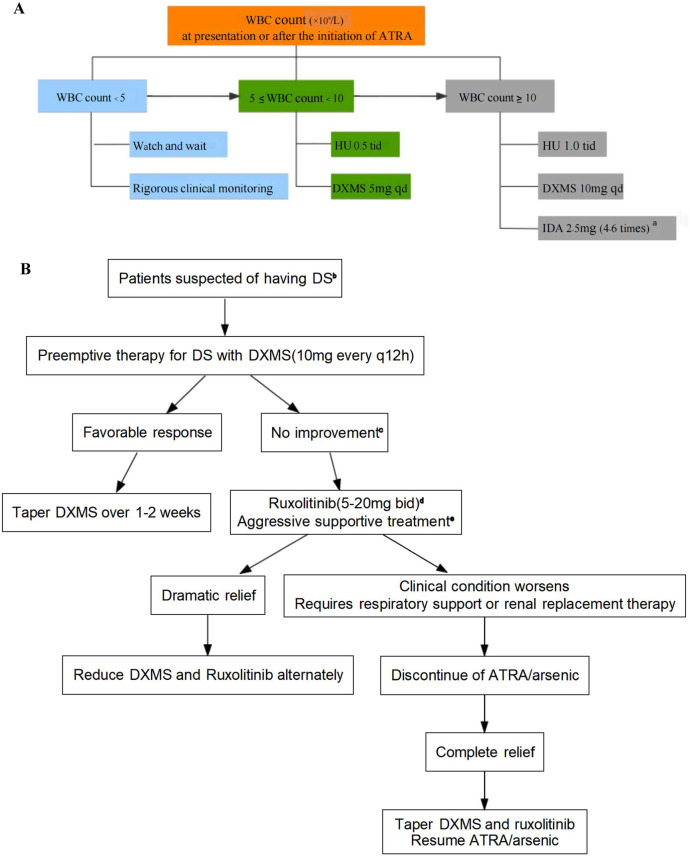
Prophylaxis and management for APL DS. **A** Prophylaxis against APL DS. **B** Management for APL DS. ^**a**^If the WBC count exceeded 10 × 10^9^/L, 4-6 doses of IDA (2-5 mg/dose) were administrated 72 hours after initiation of ATRA in case of fatal bleeding risk. ^**b**^Administrated DXMS 10 mg twice daily promptly at the earliest symptom or sign suggestive of DS, such as dyspnea, unexplained fever, weight gain greater than 5 kg, unexplained hypotension, acute renal failure, pulmonary infiltrates, or pleuropericardial effusion. ^**c**^Patients should be closely monitored. If the symptoms/signs did not improve within 24 hours or worsened in 8 hours (e.g., shortness of breath, slight hemoptysis, lower blood oxygen saturation, high-flow oxygen therapy requirement, and progressive oliguria), ruxolitinib should be initiated. ^**d**^The selection of different doses of ruxolitinib should be based on a comprehensive evaluation of the severity of DS, age, weight, general condition, and accompanying comorbidities. ^**e**^The details of aggressive supportive care are available in the Supplementary Appendix. WBC white blood cell, ATRA all-trans retinoic acid, HU hydroxyurea, DXMS dexamethasone, IDA idarubicin, DS differentiation syndrome.

When DS was suspected, prompt initiation of treatment with intravenous dexamethasone (20 mg/day) was administrated. If a favorable response was achieved, dexamethasone could be gradually tapered. However, if the symptoms/signs did not improve within 24 hours or worsened in 8 hours, ruxolitinib (5-20 mg bid) should be initiated promptly, with the dosage determined by the severity of DS, age, weight, general condition, and accompanying comorbidities. Once significant improvement was observed, the doses of dexamethasone and ruxolitinib could be alternately reduced. Conversely, if patients progressed to renal failure or respiratory failure and needed to be admitted to the Intensive Care Unit, ATRA/arsenic treatment should be discontinued. Once symptoms had completely resolved, ATRA/arsenic could be restarted (Fig. 1B). The primary study endpoint was the incidence of DS and severe DS, as well as early death. Secondary endpoints included complete remission (CR) rate, assessment of toxic effects during induction, recurrence-free survival (RFS) and overall survival (OS).

Between June 1, 2019, and December 31, 2021, 111 patients diagnosed with APL were included in this trial from 14 centers in China (Table 1). Among these patients, 33 were classified as high-risk patients (WBC count at diagnosis > 10 × 10^9^/L) and 78 as low-risk patients (WBC count at diagnosis ≤ 10 × 10^9^/L). The median WBC count before treatment in high-risk patients was 21.51 × 10^9^/L, with 10 patients having a count exceeding 50 × 10^9^/L. No patients withdrew from the study due to either treatment refusal or excessive toxicity.

**Table 1:** Characteristics of APL patients at diagnosis and comparison of the patients with and without DS, moderate DS and severe DS.

Characteristic	Total (*n* = 111, %)	Non-DS (*n* = 70, %)	DS^a^ (*n* = 41, %)	*P*	Moderate DS^b^ (*n* = 25, %)	Severe DS^b^(*n* = 16, %)	*P*
Age, y				<0.001			0.018
≤40	73 (66)	56 (80)	17 (41)		14 (56)	3 (19)	
>40	38 (34)	4 (20)	24 (59)		11 (44)	13 (81)	
Sex				0.001			0.058
Male	48 (43)	22 (31)	26 (63)		13 (52)	13 (81)	
Female	63 (57)	48 (39)	15 (37)		12 (48)	3 (19)	
BMI				0.250			0.805
Underweight	1 (1)	1 (1)	0 (0)		0	0	
Normal weight	52 (47)	28 (40)	24 (59)		14 (56)	10 (63)	
Overweight	40 (36)	28 (40)	12 (29)		7 (28)	5 (31)	
Obese	18 (16)	13 (19)	5 (12)		4 (16)	1 (6)	
ECOG				0.362			0.018
0–1	40 (36)	23 (33)	17 (41)		14 (56)	3 (19)	
≥2	71 (64)	47 (67)	24 (59)		11 (44)	13 (81)	
WBC count, x 10^9^/L				0.025			0.009
≤4	64 (58)	46 (66)	18 (44)		15 (60)	3 (19)	
>4	47 (42)	24 (34)	23 (56)		10 (40)	13 (81)	
PLT count, x 10^9^/L				0.616			0.041
≤10	13 (12)	7 (10)	6 (14)		1 (4)	5 (31)	
10–40	57 (51)	35 (50)	22 (54)		14 (56)	8 (50)	
≥40	41 (37)	28 (40)	13 (32)		10 (40)	3 (19)	
Hemoglobin, g/L				0.641			0.300
≤10	70 (63)	43 (61)	27 (66)		18 (72)	9 (56)	
>10	41 (37)	27 (39)	14 (34)		7 (28)	7 (44)	
Risk group^c^				0.101			0.249
Low-risk	78 (70)	53 (76)	25 (61)		17 (68)	8 (50)	
High-risk	33 (30)	17 (24)	16 (39)		8 (32)	8 (50)	
Fibrinogen, g/L				0.638			1.000
≤1	27 (24)	16 (23)	11 (27)		7 (28)	4 (25)	
>1	84 (76)	54 (77)	30 (73)		18 (72)	12 (75)	
D-dimer, mg/L				0.877			0.472
≤20	83 (75)	52 (74)	31 (76)		20 (80)	11 (69)	
>20	28 (25)	18 (26)	10 (24)		5 (20)	5 (31)	
LDH level, U/L				0.395			1.000
≤500	81 (73)	53 (76)	28 (68)		17 (68)	11 (69)	
>500	30 (27)	17 (24)	13 (32)		8 (32)	5 (31)	
FLT3 mutation				0.974	6/17	5/11	0.701
Yes	25 (39)	14 (39)	11 (39)		6 (35)	5 (45)	
No	39 (61)	22 (61)	17 (61)		11 (65)	6 (55)	
Cr level, ìmol/L	70.0 (57.6-82)	63.2 (54.9-72.7)	72.0 (58.5-83.0)	0.288	78.2 (59.1-86.0)	70.0 (54.2-79.9)	0.620
BM blast (%)	82.5 (20.8-97)	82.0 (29.2-118)	83.0 (20.8-97.0)	0.781	83.0 (53.0-93.8)	83.3 (20.8-97.0)	0.841

*BMI* body mass index, *BM* bone marrow, *Cr* creatinine, *DS* differentiation syndrome, *ECOG* eastern cooperative oncology group, *LDH* lactate dehydrogenase, *PLT* platelet, *WBC* white blood cell.

^a^The diagnosis of DS was made on clinical grounds by the association of at least two of the following signs, in the absence of other causes: dyspnea, unexplained fever, weight gain > 5 kg, unexplained hypotension, acute renal failure, pulmonary infiltrates or pleuropericardial effusion. A single sign or symptom of DS alone was not considered sufficient on its own to make a diagnosis of APL DS but rather served as an indication for preemptive therapy. Whenever a diagnosis of APL DS was considered, it was vital to thoroughly exclude other conditions, such as infections or heart failure, which can mimic the manifestations of DS.

^b^The patients with DS were classified into two groups: those with severe DS ( > 3 signs or symptoms aforementioned) or moderate DS (2-3 signs or symptoms aforementioned).

^c^Patients were categorized into risk groups based on their initial WBC count (≤ 10 × 10^9^/L for the low-risk APL cohort and > 10 × 10^9^/L for the high-risk APL cohort).

All high-risk patients and 57 out of 78 low-risk patients received dexamethasone (5-10 mg/day) for DS prophylaxis. Preemptive therapy with dexamethasone (10 mg every 12 hours) was administered to 65 patients (40 low-risk and 25 high-risk) due to suspected DS. Among the total 111 patients, 41 (36.9%) were ultimately diagnosed with DS, with 25 (22.5%) classified as moderate cases and 16 (14.4%) as severe cases (Table 1). The incidence of DS and severe DS was higher in high-risk patients compared to low-risk patients (48.5% vs. 32.1%, 24.2% vs. 10.3%), while these differences were not statistically significant (*p* = 0.057, *p* = 0.056) (Figure S1). DS occurred at a median of 8 days after the initiation of ATRA administration, with a range of 2-21 days. The most common features of DS included weight gain (85%), pulmonary infiltrates (78%), and dyspnea (71%). Additionally, patients with severe DS exhibited a higher prevalence of dyspnea (*p* = 0.013), pleuropericardial effusion (*p* = 0.018), and acute renal dysfunction (*p* = 0.009) compared to moderate cases (Table S1).

23 out of the 41 patients with DS experienced a rapid remission with preemptive treatment. However, the condition of the remaining patients with severe DS (12 patients) and moderate DS (6 patients) worsened (Table S2). They all received ruxolitinib, with 12 responding well, while the other 6 required non-invasive mechanical ventilation and discontinued ATRA. Five of them recovered within 72 hours, and one patient was successfully weaned from the ventilator 10 days later. None of them developed ventilator dependency or required invasive mechanical ventilation. The median duration of ruxolitinib treatment was 12.5 days, ranging from 8 to 17 days (Table S3).

In our study, 24 out of 65 patients who received preemptive therapy (17 low-risk and 7 high-risk) were ultimately not categorized as having DS. Among them, 20 cases exhibited weight gain, 2 experienced unexplained fever, and 2 presented with transient pulse oxygen decline. All patients quickly improved after receiving dexamethasone combined with supportive therapy such as diuretics, antibiotics, and oxygen inhalation. Although it cannot be completely ruled out that these patients may have exhibited early manifestations of DS, fluid overload and infection could not be ruled out either. Additionally, according to the diagnostic criteria for DS, a single sign or symptom of DS alone was not considered sufficient to make a diagnosis of DS [9]. Therefore, these 24 patients were not considered for inclusion in the DS category.

The overall 30-day mortality rate was 1.8% (2/111), with two high-risk patients succumbing to intracranial hemorrhage. There were no deaths attributed to DS or infection. The overall CR rate was 98.2%.

Early management of leukocytosis is crucial in preventing DS and typically involves the use of cytoreductive agents and steroids. In this study, our tailored treatment regimen for comprehensive prophylaxis against DS was proved effective in managing hyperleukocytosis. The incidence of DS at 36.9% was slightly higher in our study compared to previous literatures [2, 9], which could be attributed to improved recognition of the syndrome and a higher proportion (approximately 30%) of high-risk patients. Notably, unlike findings from other studies [9, 10], our results did not reveal a significant difference in the incidence of DS and severe DS among patients with different risk profiles, highlighting that high-risk patients may derive greater benefit from prophylactic measures.

Rapid resolution of symptoms was observed in only 56% of DS patients who received preemptive dexamethasone with continuous ATRA therapy. The unique characteristics of promyelocyte maturation and tissue infiltration in DS may exacerbate the cell-mediated coagulopathy of APL [9, 11], putting these individuals at a high risk of fatal hemorrhagic events. In the PETHEMA study, 61% of DS patients discontinued ATRA, with 8% of them succumbing to hemorrhage [9]. Among the 19 cases of early hemorrhagic deaths in our previous study [5], 6 patients stopped ATRA due to DS, and 3 patients with hyperleukocytosis at presentation only received ATO to mitigate DS risks. Hesitation or early suspension of ATRA due to DS increases the risk of bleeding in newly diagnosed APL patients.

In some cases, DS can still manifest as a fulminant course even after discontinuing ATRA. Although accompanied by typical DS symptoms, hemoptysis in these patients is usually believed to be caused by coagulation disorders. However, Nicolls et al [12] identified diffuse alveolar hemorrhage as a rare symptom of DS resulting from endothelial damage in 1998. In our study, four out of the five patients with hemoptysis (cases 2, 6, 7, and 40) were also developing DS simultaneously (Table S4). When compared with patients with intracranial hemorrhage, three patients with hemoptysis showed significant improvement in coagulopathy, they recovered only after ruxolitinib was initiated. It is conceivable that hemoptysis may manifest as a result of lung injury in DS.

Although the exact pathogenesis of DS is unknown, differentiation therapy may induce the production of chemokines in the lung and in APL cells, both of which trigger the migration of leukemic cells. Pulmonary infiltration of activated leukocytes may induce an uncontrollable systemic hyperinflammatory reaction known as cytokine release syndrome (CRS) [13]. Therefore, we hypothesized that interventions such as the selective JAK1/2 inhibitor, ruxolitinib, aimed at modulating the immune response and suppressing exacerbating hyperinflammation might help resolve this complex issue.

The efficacy of ruxolitinib for steroid-refractory DS was 67% when ATRA was maintained. After discontinuing ATRA therapy, the remaining patients recovered. In contrast, DS-associated mortality was 8%-11% in other trials [9, 10, 14]. Furthermore, in the safety analysis (Supplementary Appendix), due to progressive tapering of the doses within a brief course and minimal chemotherapy, the incidence of grade 4 neutropenia did not differ significantly between patients with or without DS. Infection occurred in 40% of all patients in this study, a lower rate compared to patients in the APL15 trial [15], using ATRA-ATO induction without chemotherapy (50%).

In conclusion, our data support the feasibility and benefits of the personalized strategies including steroids and ruxolitinib for managing DS in APL, resulting in a reduction of early death.

### Supplementary information

Supplementary Appendix

## Data Availability

The data that supports the findings of this study are available in the supplementary material of this article.
