# Supplementary material for: Improved prevention and treatment strategies for differentiation syndrome contribute to reducing early mortality in patients with acute promyelocytic leukemia
Source: Blood Cancer J. 2024 Jul 15;14(1):113. doi: 10.1038/s41408-024-01074-y (PMC11251030; doi:10.1038/s41408-024-01074-y)
Supplement: Supplementary file 1 — Supplementary Appendix [file 41408_2024_1074_MOESM1_ESM.docx]

Supplementary Appendix

This appendix has been provided by the authors to give readers additional information about their work.

Supplement to: Qian Wu, Xiaofei Yang, Jingren Zhang, et al. Improved Prevention and Treatment Strategies for Differentiation Syndrome Contribute to Reducing Early Mortality in Patients With Acute Promyelocytic Leukemia

TABLE OF CONTENTS

[Methods 1](#_Toc30788)

Study definition, monitoring, supportive measures and management of complications

[3](#_Toc469)

[Statistical methods 5](#_Toc26215)

[Results 6](#_Toc27668)

[Treatment Toxicities 8](#_Toc23378)

[Tables S1-S5 9](#_Toc32121)

[Figures S1-S2 1](#_Toc13645)5

**Methods**

**Patients**

Eligibility criteria included age of 18 to 75 years, a diagnosis of acute promyelocytic leukemia (APL) with t (15; 17) or PML/RARα rearrangement, Q-Tc interval less than 500 ms, absence of serious comorbidity, and an Eastern Cooperative Oncology Group (ECOG) performance status between 0 and 4.

**Study design**

In the prospective Phase 3 multicenter single-arm APL-01 trial, all patients diagnosed with APL, regardless of white blood cell (WBC) counts, received induction treatment with all-trans retinoic acid (ATRA) in combination with either intravenous arsenic trioxide (ATO) or oral tetra-arsenic tetra-sulfide (As4S4) formula named the Realgar-Indigo naturalis formula (RIF) [8].

**Induction treatment**

Treatment with ATRA at a dose of 25 mg/m^2^ and blood product support was initiated upon suspicion of APL, depended on morphology, and/or coagulopathy as indicated by a positive disseminated intravascular coagulation screen. Once a positive result of a t (15; 17) translocation and/or PML-RARα rearrangement was confirmed, ATO (0.16 mg/kg capped at 10 mg) or RIF (60 mg/kg) was administered until complete remission (CR) was achieved. ATRA treatment was to be discontinued if the diagnosis was not supported by genetic or molecular data.

**Prophylaxis for APL DS**

The prevention regimen for differentiation syndrome (DS) was based on the WBC count at presentation and after the initiation of ATRA, which included cytoreductive agents and dexamethasone (Fig 1a**)**. For patients with a WBC count between 5 and 10 × 10^9^/L, hydroxyurea (500 mg three times a day) and dexamethasone (5 mg/day) were administered. If the WBC count increased to greater than 10 × 10^9^/L, the dose of hydroxyurea was increased to 1000 mg three times a day, dexamethasone was increased to 10 mg/day, and 4-6 doses of IDA (2-5 mg/dose) were administrated 72 hours after initiation of ATRA in case of fatal bleeding risk. These medications were then gradually tapered and discontinued once the WBC count dropped to less than 10 × 10^9^/L.

**Management for APL DS**

When DS was suspected, prompt initiation of treatment with intravenous dexamethasone at a dose of 20 mg per day was recommended. Patients should be closely monitored for volume overload (daily intake and output), weight and pulmonary status, creatinine level, lactate dehydrogenase (LDH level), and cytokine level. If a favorable response was achieved, dexamethasone could be gradually tapered over a period of 1-2 weeks. If these symptoms/signs did not improve within 24 hours or worsened in 8 hours (e.g., shortness of breath, slight hemoptysis, lower blood oxygen saturation, high-flow oxygen therapy requirement, and progressive oliguria), ruxolitinib should be initiated at a dose of 5-20 mg twice a day, with the dosage determined by the severity of DS, age, weight, general condition, and accompanying comorbidities. For example, an older patient with moderate DS and several complications would initially receive a dosage of 5 mg twice daily, which could be increased up to 20 mg twice daily if necessary. Conversely, if a younger patient had rapidly progressing and severe DS, the dosage could be started at 20 mg twice daily immediately. Once significant relief was observed, the doses of dexamethasone and ruxolitinib could be alternately reduced. If patients progressed to renal failure or respiratory failure and were admitted to the Intensive Care Unit, ATRA/arsenic agent treatment should be discontinued. Once signs and symptoms of APL DS had completely resolved, ATRA/arsenic agent could be restarted (Fig 1b).

**Study definition, monitoring, supportive measures and management of complications**

**Definition**

Differentiation syndrome (DS). The diagnosis of DS was made based on clinical grounds by the presence of at least two of the following signs, in the absence of other causes: dyspnea, unexplained fever, weight gain > 5 kg, unexplained hypotension, acute renal failure, pulmonary infiltrates or pleuropericardial effusion. The PETHEMA group defined the grading of DS and classified patients as having severe DS (>3 signs or symptoms) or moderate DS (2-3 signs or symptoms) [9]. When considering a diagnosis of APL DS, it was vital to thoroughly exclude other conditions, such as infections or heart failure, that could mimic the manifestations of DS.

Early death. Early death referred to any deaths occurring from the initiation of therapy until the end of induction therapy, which could sometimes extend up to 30 days.

Complete remission (CR). CR was defined as a patient having less than 5% blasts and atypical promyelocytes in an aspirate sample, an absolute neutrophil count of more than 10^9^/L, and a Platelet (PLT) count of more than 100 × 10^9^/L.

Overall survival (OS). OS was defined as the time from entry into the study until death from any cause, with patients alive at the last follow-up being censored.

Recurrence-free survival (RFS). RFS was defined for patients who achieve CR and was measured from the date of attaining the leukemia-free state until the date of APL relapse or death from any cause, whichever occurred first.

Toxic effects. Toxic effects were graded according to the Terminology Criteria for Adverse Events, version (CTCAE V5.0).

**Monitoring：**

WBC and PLT counts, as well as routine coagulation parameters such as prothrombin time (PT), activated partial thromboplastin time (APTT), thromboplastin time (TT) and fibrinogen (Fbg) should be monitored at least daily, and more frequently if necessary.

An electrocardiogram (ECG) should be conducted to assess prolonged QTc interval and optimize serum electrolytes (calcium, potassium, magnesium) for the safe administration of arsenic.

**Supportive measures and management of complications**

Transfusions of blood products should aim to maintain the fibrinogen concentration above 1500mg/L, the PLT count above 50×10^9^/L, and PT and APTT levels close to normal. Transfusions should be continued during induction therapy until there was no evidence of coagulopathy in both clinical and laboratory findings. To address the issue of fluid overload in patients with poor diuretic effects, we considered partially replacing fresh frozen plasma with cryoprecipitate and human fibrinogen.

The management of all APL patients required aggressive supportive care, especially for patients with DS. Fluid overload and gradual weight gain should be aggressively treated with diuretics, and if severe volume overload and renal dysfunction occurred, renal replacement therapy might be necessary. Additionally, invasive and noninvasive mechanical ventilation might be indicated in some patients with severe acute respiratory failure who did not respond to high-flow oxygen therapy.

Furthermore, any other suspected complications, such as infection, cardiac insufficiency, or embolism, should be complemented with appropriate treatments.

**Statistical methods**

The distributions of continuous data were examined using the Kolmogorov-Smirnov test. Skewed data were presented as the median and range, and the differences were compared using the Mann-Whitney U test. Categorical data were presented as sample size and percentage, and the differences were compared using the chi-square test (or Fisher's exact test). Subsequently, multivariate analysis (automatic selection model) was performed for variables with a p-value less than 0.1 in the univariate analysis. The Kaplan-Meier test was used to compare OS between the high-risk and low-risk patients, as well as between DS and Non-DS groups. All statistical analyses were conducted using SPSS 23.0 software (SPSS Inc., Chicago, IL, USA), with a p-value less than 0.05 considered statistically significant.

**Results**

All the patients in the high-risk group and 57 out of 78 (73.1%) patients in the low-risk group received dexamethasone （5-10mg/d） as prophylaxis for DS according to our protocol. Additionally, 40 out of 78 low-risk patients (51.3%) and 25 out of 33 high-risk patients (75.8%) received preemptive therapy with dexamethasone （10mg q12h）due to suspected DS.

**Predictive factors for DS and severe DS**

Upon univariate analysis of prognostic factors (Table 1), it was found that age older than 40 years (*p* < 0.001), male gender (*p* = 0.001) and WBC count exceeding 4 × 10^9^/L (*p* = 0.025) were associated with developing DS. Following multivariate analysis (Table S5), only a WBC count greater than 4 × 10^9^/L (*p* < 0.001) remained statistically significant.

Regarding severe DS, univariate analysis (Table 1) identified predictive factors such as age older than 40 years (*p* = 0.018), ECOG score ≥ 2 (*p* =0.018), a WBC count exceeding 4 × 10^9/^L (*p* = 0.009), and PLT (＜ 10 × 10^9^/L) (*p* = 0.041). Male gender displayed a marginal significant difference between the two groups (*p* = 0.058). However, following multivariate analysis (Table S5), only advanced age (＞ 40 years old）(*p* = 0.015) maintained its predictive value.

**Clinical outcomes**

The overall 30-day mortality rate was 1.8% (2/111), with two high-risk patients succumbing to intracranial hemorrhage, one on day 8 and the other on day 9 after initiation of ATRA. There were no deaths attributed to DS or infection. Out of the 111 patients, the overall CR rate was 98.2%. Specifically, 100% (78/78) of the low-risk patients and 93.9% (31/33) of the high-risk patients achieved CR. The median time interval for achieving CR was 26 days (range: 18 to 71 days) in the overall analysis. Significant differences were observed in the median time to CR between high-risk patients and low-risk patients (29 days vs. 26 days; *p* = 0.001), as well as between the DS group and the Non-DS group (31 days vs. 25 days; *p* = 0.000).

Our trial demonstrated a 100% negative rate for RQ-PCR among the remaining 109 evaluable patients at 3 months after diagnosis. All patients completed 7 cycles of consolidation therapy, with RIF administered at 60 mg/kg daily in a 4-week-on and 4-week-off regimen for 4 cycles, and ATRA at a dose of 25 mg/m^2^ daily in a 2-week-on and 2-week-off regimen for 7 cycles. However, patients with high-risk APL required 9 cycles of maintenance therapy with ATRA at 25 mg/m^2^ daily for 2 weeks every 4 weeks on cycles 1, 4, 7 and RIF at 60 mg/kg daily for 2 weeks every 4 weeks on cycles 2, 3, 5, 6, 8, 9.

The present analysis was carried out in September 2023, with a median follow-up of 34 months. Four patients (3 low-risk and 1 high-risk) experienced relapse, with three experiencing hematological relapse in the 11th, 16th, and 25th months, and one developing central nervous system leukemia in the 17th month. Among these patients, one died of intracranial hemorrhage, while the others eventually achieved CR2 and survived.

The 3-year OS and RFS rates did not exhibit a significant difference between high-risk and low-risk patients with rates of 93.9% versus 98.7% （*p* = 0.196; Fig S2a) and 90.9% versus 96.0%（*p* = 0.265; Fig S2b), respectively. Additionally, there was no statistically significant difference in OS between the DS and Non-DS groups (Fig S2c). The 3-year RFS rates in patients with and without DS were 97.6% and 92.7% （*p* = 0.322; Fig S2d).

**Treatment Toxicities**

During induction therapy, 51 patients (46%) developed grade 4 neutropenia, with eight patients (7.2%) experiencing agranulocytosis lasting more than 14 days; only two of them had received ruxolitinib. High-risk patients were more likely to develop grade 4 neutropenia compared to low-risk patients (22/33 vs. 29/78, *p* < 0.05). There was no significant difference between patients with DS and those without DS (23/41 vs. 28/70, *p* ＞ 0.05).

Furthermore, 44 infectious events occurred in 42 patients (40%) during the induction period, including 25 cases of pneumonia, 6 cases of upper respiratory tract infection, 6 cases of intestinal infection, 2 cases of sepsis, 2 cases of gingivitis, 2 cases of soft tissue infection, and 1 case of cholecystitis. Severe bleeding was observed in 22 patients, including intracranial hemorrhage in 7 patients, hemoptysis in 5 patients, gastrointestinal bleeding in 4 patients, hematuria in 6 patients, retinal hemorrhage in 3 patients, and corpus luteum rupture in 1 patient. Additional information about these patients can be found in Table S4. At the time of bleeding, only 27% of patients reached the target PLT count (50 × 109/L), and 40% achieved the target fibrinogen level (1.5 g/L). One high-risk patient experienced a cerebral infarction upon presentation. Three low-risk patients developed thrombosis, with one patient experiencing a pulmonary embolism on day 22, while two patients had catheter-related thrombosis on day 22 and day 25, respectively.

Thirty-one patients (28%) experienced grade 3 or 4 hepatic toxic effects. In all cases, liver function abnormalities resolved upon temporary discontinuation of agents such as arsenic, triazole antifungals, and norethisterone. No of the patients experienced grade 3 or 4 prolonged QTc intervals. Transient hyperglycemia (8.1%), likely due to dexamethasone treatment, occurred in nine patients without a history of diabetes. All of them successfully recovered with hypoglycemic therapy.

**Supplementary Table 1.** Clinical characteristics of APL patients with DS.

| Characteristic | DS  (n=41, %) | Moderate DS (n=25, %) | Severe DS (n=16, %) | χ2 | P |
| --- | --- | --- | --- | --- | --- |
| Dyspnea | 29 (71) | 14 (56) | 15 (94) | NA | 0.013 |
| Unexplained fever | 14 (34) | 6 (24) | 8 (50) | 2.933 | 0.087 |
| Weight gain^a^ | 35 (85) | 20 (80) | 15 (94) | NA | 0.376 |
| Pulmonary infiltrates | 32 (78) | 18 (72) | 14 (88) | NA | 0.441 |
| Pleuropericardial effusion | 24 (59) | 11 (44) | 13 (81) | 5.577 | 0.018 |
| Acute renal dysfunction | 7 (17) | 1 (4) | 6 (38) | NA | 0.009 |
| Unexplained hypotension | 2 (5) | 0 | 2 (13) | NA | 0.146 |

*DS* differentiation syndrome, *NA* not available.

a. We observed weight gain of more than 5kg in 55 patients, all of whom received preemptive therapy with dexamethasone and diuretics. Among them, 20 patients solely experienced weight gain without any other typical manifestations of DS. As a single sign or symptom of DS alone is not adequate for diagnosing APL-DS, these patients were not classified as having DS.

**Supplementary Table 2.** Effectiveness of the management of DS.

| Treatment | | | | | Low-risk, n=78 | | High-risk, n=33 | | Total, n=111 | |
| --- | --- | --- | --- | --- | --- | --- | --- | --- | --- | --- |
|  |  |  |  |  | n | % | n | % | n | % |
| Prophylaxis for APL DS | | | | | 57 | 73 | 33 | 100 | 90 | 81 |
| Preemptive therapy （DXMS 10mg q12h） | | | | | 40 | 51 | 25 | 76 | 65 | 59 |
|  | Definite diagnosis for APL DS | | | | 25 | 32 | 16 | 49 | 41 | 37 |
|  |  | Moderate DS | | | 17 | 22 | 8 | 24 | 25 | 23 |
|  |  |  | Favorable response to DXMS | | 13 |  | 6 |  | 19 |  |
|  |  |  | No improvement: Ruxolitinib | | 4 |  | 2 |  | 6 |  |
|  |  |  |  | Dramatic relief | 4 |  | 2 |  | 6 |  |
|  |  |  |  | Worsening: stop ATRA | 0 |  | 0 |  | 0 |  |
|  |  | Severe DS | | | 8 | 10 | 8 | 24 | 16 | 14 |
|  |  |  | Favorable response to DXMS | | 3 |  | 1 |  | 4 |  |
|  |  |  | No improvement: Ruxolitinib | | 5 |  | 7 |  | 12 |  |
|  |  |  |  | Dramatic relief | 3 |  | 3 |  | 6 |  |
|  |  |  |  | Worsening: stop ATRA | 2 |  | 4 |  | 6 |  |

*DS* differentiation syndrome, *DXMS* dexamethasone, *ATRA* all-trans retinoic acid.

**Supplementary Table 3.** Effectiveness of the management of DS

| **UPN** | **Risk-**  **group** | **Grading of severity DS** | **DXMS treatment** | | **Ruxolitinib treatment** | | | | **ATRA treatment** | |
| --- | --- | --- | --- | --- | --- | --- | --- | --- | --- | --- |
|  |  |  | Initiation date**^a^** | Duration  （days） | Initiation date**^a^** | Initial dose | Evaluation | Duration  （days） | Hold/kept | Restart date**^a^** |
| 2 | High | Severe | Day 10 | 12 | Day 10 | 20mg bid | Progression | 11 | Hold (Day10) 10101010) | Day 18 |
| 6 | High | Severe | Day 8 | 18 | Day 8 | 20mg bid | Progression | 17 | Hold (Day 8) | Day 19 |
| 7 | High | Severe | Day 21 | 15 | Day 21 | 15mg bid | Progression | 16 | Hold (Day21) | ND |
| 10 | High | Moderate | Day 8 | 16 | Day 10 | 5mg bid | Relief | 15 | Kept |  |
| 35 | Low | Moderate | Day 7 | 11 | Day 7 | 10mg bid | Relief | 10 | Kept |  |
| 40 | Low | Severe | Day 4 | 14 | Day 7 | 15mg bid | Relief | 12 | Kept |  |
| 42 | Low | Severe | Day13 | 9 | Day13 | 10mg bid | Relief | 8 | Kept |  |
| 44 | Low | Severe | Day 6 | 13 | Day 6 | 15mg bid | Progression | 14 | Hold (Day 6) | Day 18 |
| 47 | Low | Moderate | Day 9 | 13 | Day 10 | 10mg bid | Relief | 11 | Kept |  |
| 72 | Low | Severe | Day 5 | 11 | Day 5 | 15mg bid | Relief | 9 | Kept |  |
| 74 | High | Severe | Day 10 | 13 | Day 10 | 20mg bid | Progression | 13 | Hold (Day10) | Day 18 |
| 91 | Low | Severe | Day 9 | 10 | Day 10 | 20mg bid | Relief | 9 | Kept |  |
| 93 | High | Severe | Day 4 | 14 | Day 4 | 20mg bid | Relief | 13 | Kept |  |
| 94 | Low | Moderate | Day 10 | 11 | Day 10 | 10mg bid | Relief | 10 | Kept |  |
| 99 | High | Severe | Day 8 | 14 | Day 9 | 10mg bid | Relief | 14 | Kept |  |
| 102 | High | Moderate | Day 4 | 12 | Day 4 | 20mg bid | Relief | 13 | Kept |  |
| 105 | High | Severe | Day 4 | 14 | Day 4 | 10mg bid | Progression | 13 | Hold (Day5) | Day15 |
| 109 | Low | Moderate | Day 15 | 10 | Day 16 | 10mg bid | Relief | 10 | Kept |  |

*DS* differentiation syndrome, *UPN* unique patient number, *DXMS* dexamethasone, *ATRA* all-trans retinoic acid, *ND* not done.

1. Therapy with ATRA was started.

**Supplementary Table 4**. Details of patients who experienced severe hemorrhage during remission induction course.

| **UPN** | **Risk-**  **group** | **Onset of hemorrhage** | | | | | | **DS** | | **Outcome** |
| --- | --- | --- | --- | --- | --- | --- | --- | --- | --- | --- |
|  |  | Date**^a^** | Site of bleeding | WBC count (10^9^/L) | PLT count (10^9^/L) | D-dimer (mg/L) | Fbg (g/L) | Date**^a^** | Grading of severity |  |
| 2 | High | Day 10 | Lung | 49.4 | 60 | 15.52 | 1.88 | Day 10 | Severe | Alive |
| 6 | High | Day 8 | Lung | 71.81 | 47 | 34.42 | 1.29 | Day 8 | Severe | Alive |
| 7 | High | Day 22 | Lung | 2.68 | 15 | 5.4 | 0.88 | Day 21 | Severe | Alive |
| 9 | High | Day 10 | Urinary system | 83.31 | 17 | > 20 | 0.94 | Day 8 | Severe | Alive |
| 10 | High | Day 5 | Urinary system | 136.22 | 47 | > 20 | 0.99 | Day 8 | Moderate | Alive |
| 13 | High | Day 7 | Gastrointestine, Urinary system | 262.17 | 52 | 46.26 | 2.37 | Day 6 | Moderate | Alive |
| 16 | High | Day 7 | Retina | 46.31 | 54 | ND | 1.47 | Day 6 | Moderate | Alive |
| 18 | High | Day16 | Retina | 9.6 | 43 | 1.48 | 2.64 | - | - | Alive |
| 27 | High | Day 9 | CNS, Urinary system, Retina | 117.87 | 33 | > 20 | 1.13 | - | - | ED |
| 31 | High | Day 8 | CNS, Gastrointestine | 243.83 | 62 | 6.05 | 1.35 | - | - | ED |
| 34 | High | Day 0 | CNS | 20.87 | 8 | >20 | 1.02 | Day 6 | Moderate | Alive |
| 40 | Low | Day 7 | Lung | 52.84 | 34 | 53.04 | 2.04 | Day 4 | Severe | Alive |
| 42 | Low | Day 18 | Lung | 1.53 | 28 | > 20 | 0.78 | Day 13 | Severe | Alive |
| 43 | Low | Day 21 | Gastrointestine | 4.52 | 11 | 52.99 | 0.6 | Day 9 | Severe | Alive |
| 45 | Low | Day 0 | Urinary system | 1.2 | 102 | 1.12 | 0.8 | Day 10 | Severe | Alive |
| 47 | Low | Day 2 | Urinary system | 34.67 | 15 | ND | 1.71 | Day 9 | Moderate | Alive |
| 52 | Low | Day 0 | Gastrointestine | 7.6 | 49 | > 20 | 0.96 | Day 12 | Moderate | Alive |
| 70 | Low | Day 0 | CNS | 1.05 | 23 | > 20 | 0.97 | - | - | Alive |
| 100 | Low | Day 0 | CNS | 4.01 | 16 | > 20 | 2.58 | - | - | Alive |
| 104 | Low | Day 0 | Corpus luteum | 2.17 | 37 | 10.9 | 2.02 | - | - | Alive |
| 107 | Low | Day 3 | CNS | 5.6 | 12 | 6.76 | 4.72 | Day 5 | Moderate | Alive |
| 110 | Low | Day 0 | CNS | 3.89 | 3 | ND | 0.99 | Day 13 | Moderate | Alive |

*UPN* unique patient number, *CNS* central nervous system, *WBC* white blood cell, *PLT p*latelet, Fbg fibrinogen, *ND* not done, *ED* early death.

a. Therapy with ATRA was started.

**Supplementary Table 5.** Multivariate analysis of different risk and DS stages for APL patients.

|  | **χ2** | **OR (95% CI)** | **P** |
| --- | --- | --- | --- |
| **DS vs. Non-DS** |  |  |  |
| Age (Reference category: ≤ 40) | 2.311 | 0.958 (0.907-1.012) | 0.128 |
| Sex (Reference category: Male) | 1.011 | 0.492 (0.123-1.963) | 0.315 |
| WBC (Reference category: < 4) | 20.805 | 1.241 (1.131, 1.361) | <0.001 |
| **Severe DS vs. Moderate DS** |  |  |  |
| Age (Reference category: ≤ 40) | 5.968 | 12.200 (1.640-90.772) | 0.015 |
| Sex (Reference category: Male) | 1.163 | 0.342 (0.049-2.406) | 0.281 |
| ECOG (Reference category: ≤ 1) | 0.396 | 1.374 (0.511-3.694) | 0.529 |
| WBC (Reference category: < 4) | 2.891 | 2.019 (0.898-4.539) | 0.089 |
| PLT (Reference category: < 10) | 1.351 | 0.426 (0.101-1.797) | 0.245 |

*BM* bone marrow, *CI* confidence interval, *DS* differentiation syndrome, *LDH* lactate dehydrogenase, *OR* odds ratio, *PLT* platelet, *WBC* white blood cell


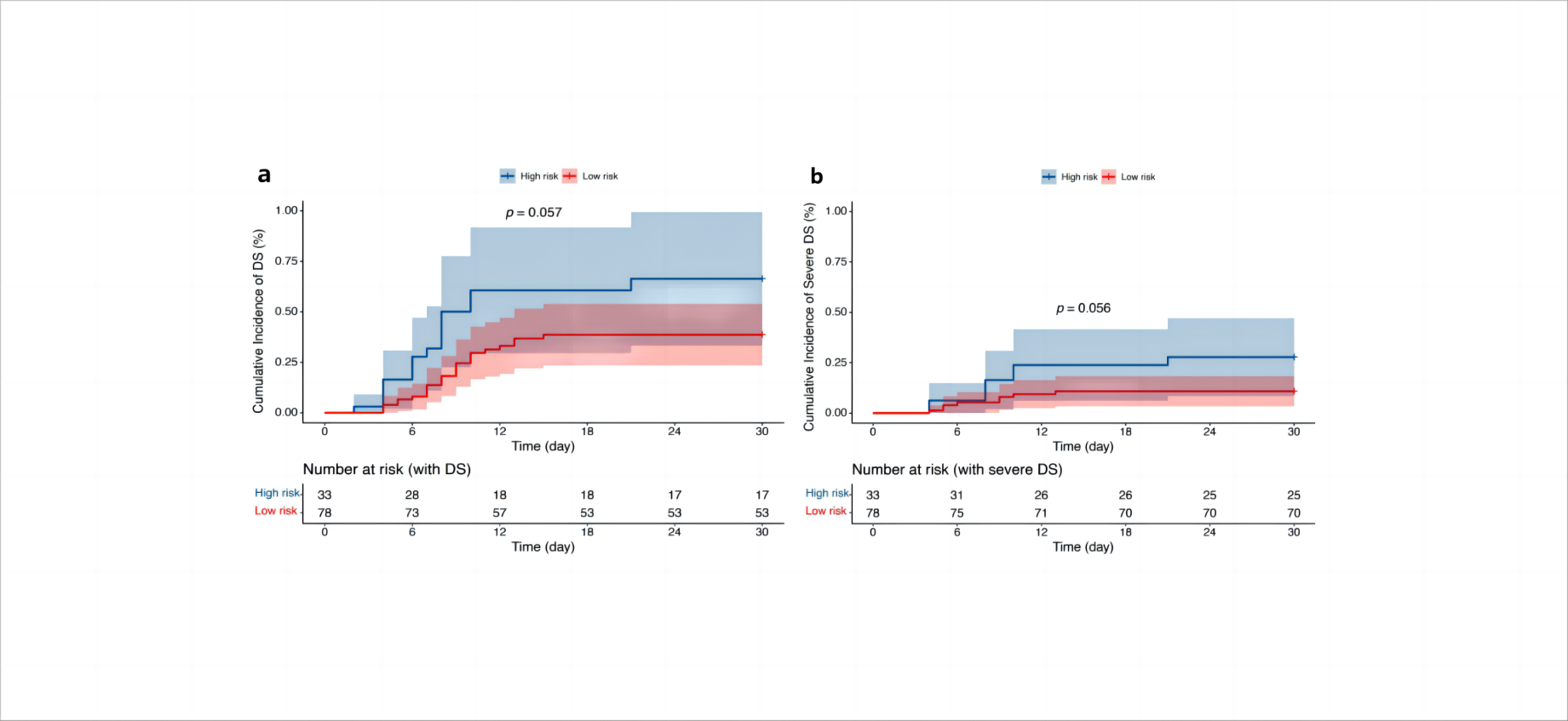


*DS* differentiation syndrome.

**Figure S1.** The 30-day cumulative incidence rates of DS and severe DS. **a** The incidence of DS in the low-risk and high-risk groups. **b** The incidence of severe DS in the low-risk and high-risk groups.


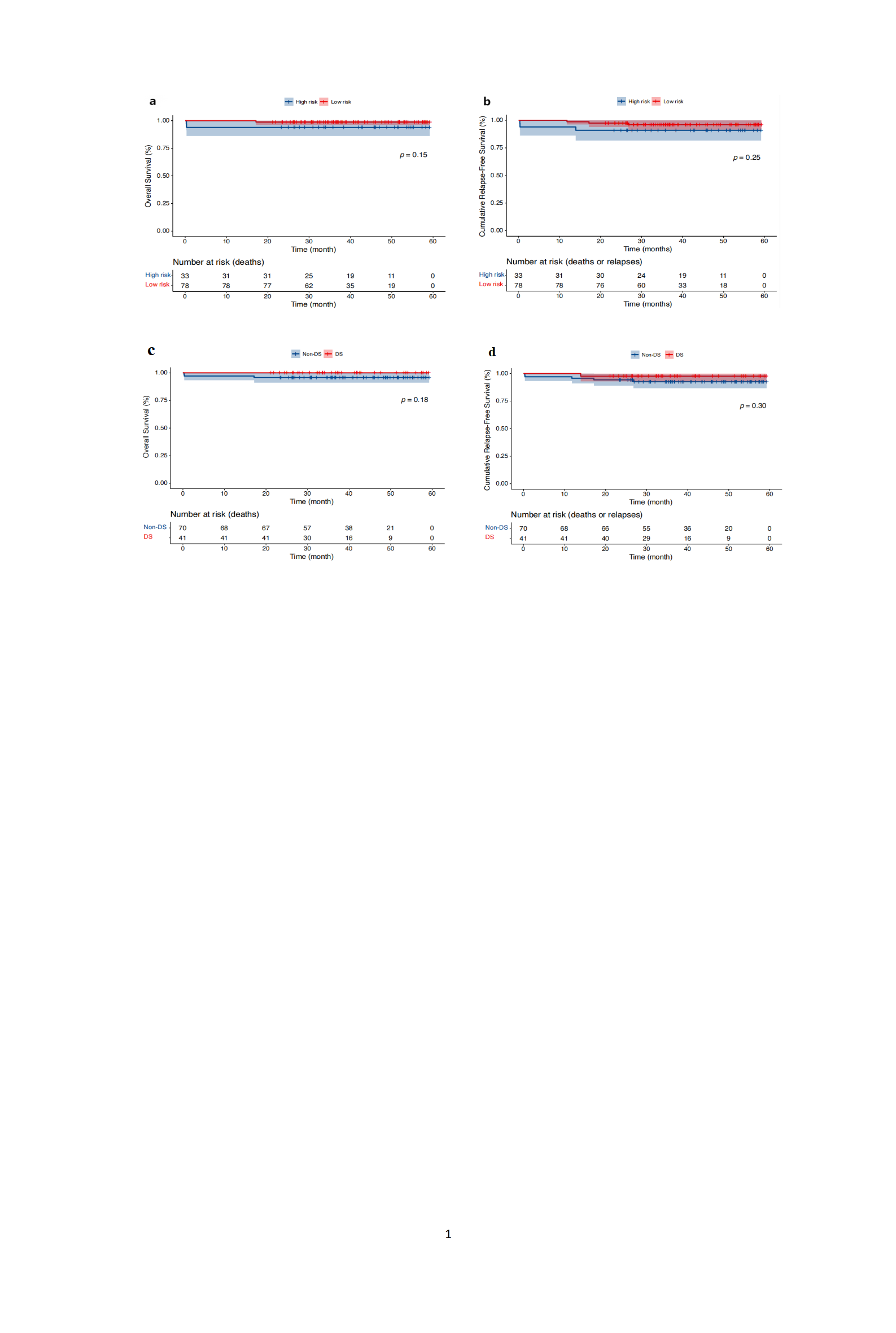


*DS* differentiation syndrome, *RFS* recurrence-free survival, *OS* overall survival.

**Figure S2.** The 3-year OS and RFS rates between the high-risk and low-risk groups, DS and Non-DS groups. **a - b** Compared survival outcomes among patients with high-risk and low-risk APL. **c - d** Compared survival outcomes among APL patients with or without DS.

**c**
